# Detection of epidermal growth factor receptor mutations in formalin fixed paraffin embedded biopsies in Malaysian non-small cell lung cancer patients

**DOI:** 10.1186/1423-0127-20-22

**Published:** 2013-04-16

**Authors:** Tiffany Ng Shi Yeen, Rajadurai Pathmanathan, Mohd Sidik Shiran, Fattah Azman Ahmad Zaid, Yoke Kqueen Cheah

**Affiliations:** 1 Department of Biomedical Sciences, Faculty of Medicine and Health Sciences, Universiti Putra Malaysia, 43400, Serdang, Selangor, Malaysia; 2 Monash University, Jalan Lagoon Selatan, Bandar Sunway 46150Selangor, Malaysia; 3 Department of Pathology, Faculty of Medicine and Health Sciences, Universiti Putra Malaysia, 43400, Serdang, Selangor, Malaysia; 4 Department of Community Health, Faculty of Medicine and Health Sciences, Universiti Putra Malaysia, 43400, Serdang, Selangor, Malaysia

**Keywords:** EGFR, Scorpion ARMS, HRM

## Abstract

**Background:**

Somatic mutations of the epidermal growth factor receptor (*EGFR*) are reportedly associated with various responses in non-small cell lung cancer (NSCLC) patients receiving the anti-*EGFR* agents. Detection of the mutation therefore plays an important role in therapeutic decision making. The aim of this study was to detect *EGFR* mutations in formalin fixed paraffin embedded (FFPE) samples using both Scorpion ARMS and high resolution melt (HRM) assay, and to compare the sensitivity of these methods.

**Results:**

All of the mutations were found in adenocarcinoma, except one that was in squamous cell carcinoma. The mutation rate was 45.7% (221/484). Complex mutations were also observed, wherein 8 tumours carried 2 mutations and 1 tumour carried 3 mutations.

**Conclusions:**

Both methods detected *EGFR* mutations in FFPE samples. HRM assays gave more *EGFR* positive results compared to Scorpion ARMS.

## Background

Lung cancer is the main cause of cancer-related death worldwide, with over one million deaths per year [1]. Lung carcinoma is divided into two groups – non-small cell lung cancer (NSCLC) and small cell lung cancer (SCLC), based on its clinical and histopathological features. The NSCLC accounts for about 80% of lung cancer and can be further divided into three subclasses: adenocarcinoma; squamous cell carcinoma; and large cell carcinoma. In Malaysia, of all the cancer cases, lung cancer ranked second in males and sixth in females [2]. Despite advances in molecular pathology and improvement in screening programs, patients’ prognosis remains poor. Most lung cancer patients are diagnosed in the advanced or metastatic stages with a median survival of about 4–5 months while the 1-year survival rate is less than 10%, if left untreated [3].

Epidermal growth factor receptor (*EGFR*) is a transmembrane glycoprotein encoded by a gene located at the short arm of chromosome 7. Activation of the receptor through the binding of a ligand stimulates a range of cellular functions such as cell proliferation, differentiation, adhesion, migration and survival. Mutation in the *EGFR* would result in continuous tyrosine kinase activity regardless of the presence of stimulus which in turn, leads to the development of lung tumours [4]. In 2004, *EGFR* mutations in NSCLC were discovered to be associated with patients’ responsiveness to epidermal growth factor receptor (*EGFR*) tyrosine kinase inhibitors (TKI) [5-7]. Since then, *EGFR* mutation has become an important biomarker in lung cancer screening as identifying this biomarker can predict which patient will benefit from *EGFR* targeted therapy [8].

Previous studies showed that *EGFR* mutations are more common in tumours from female patients, Asian origin, never-smoker and adenocarcinoma histology [6,9]. These mutations were reported to be found in exon 18 to 21 located in the intracellular TK-containing domain. Approximately 90% of the mutations were detected in the following two hotspots: in-frame deletions in exon 19 and a missense mutation at codon 858 (*L858R*) in exon 21 [7,10,11]. These mutations are often termed classical activating mutations [12]. Besides classical mutations, other non-classical mutations in exon 18 to 21 have also been reported. They include point mutations in exon 18 (*G719X*) and exon 21 (*L861Q*), as well as substitution mutation (*S768I*) and insertions in exon 20. It is uncommon to detect non-classical mutations and patients harbouring these mutations have variable responses to *EGFR* TKIs. Moreover, there were cases of complex mutations pattern whereby two or more concomitant sites of *EGFR* mutations co-exist within a single patient [13-15].

Because of the high rates of *EGFR* mutation in Asian populations, routine *EGFR* mutation testing is essential to identify which patient will benefit from the *EGFR* targeted therapy. Direct sequencing has been the most widely used method in *EGFR* mutation testing as this method has the capability of detecting all mutations, both known and unknown. However, its time consuming and limited sensitivity due to contamination of non-malignant cells in samples render researchers to look for alternative testing methods that are faster and more sensitive [16,17].

The Scorpion Amplification Refractory Mutation System (ARMS) combines two technologies, namely ARMS and Scorpion, to detect EGFR mutations in real-time PCR reactions. ARMS allows allele specific amplification while Scorpion molecules which consist of PCR primer, covalently linked to a probe held in a hairpin loop conformation by the presence of complementary stem sequence at the 5′ and 3′ ends. When both technologies are used in combination, a highly sensitive and fast method in single-base mutation detection was achieved [18,19].

Using the Scorpion ARMS kit and HRM assay, we aim to detect major *EGFR* mutations and to determine the reliability between the two methods in *EGFR* mutation detection.

## Methods

### Patients

Tumour samples, in the form of unstained sections from formalin fixed, paraffin-embedded (FFPE) tissue block, from patients with NSCLC were received in Sime Darby Medical Centre, Subang Jaya, Malaysia. The samples were assessed by pathologists from Sime Darby Medical Centre prior to the testing. A total of 484 patients were recruited for this study. Of the 484 samples, 467 were adenocarcinoma, 12 were squamous cell carcinoma, 3 were large cell carcinoma, and 2 were of other histology. This study was approved by Sime Darby independent ethics committee (IRB reference number: 201102.3), by Universiti Putra Malaysia ethics committee (Reference number: UPM/FPSK/PADS/T7-MJKEtikaPer/F01 (JSB_Aug(11)03)) and National Institute of Health, Malaysia (Date: 12-07-2011).

### DNA extraction

Genomic DNA was isolated from FFPE tissue section using QIAamp DNA FFPE Tissue Kit (QIAGEN) according to the manufacturer’s instructions. Extracted DNA was spectrophotometrically quantified using NanoPhotometer (Implen) and was stored at −20°C until use.

### EGFR mutation detection

EGFR PCR Kit (QIAGEN Manchester Ltd., United Kingdom), which combined two technologies, the Amplification Refractory Mutation System (ARMS) and Scorpion, was used to detect mutations in real-time PCR reactions. All reactions were done in 25 μl volumes using 5 μl of template DNA, 16 μl of reaction mix, 0.2 – 0.8 μl of Taq polymerase and PCR grade water.

### HRM assays

PCR for HRM analysis was performed in 0.2 ml tubes on the Rotor-Gene 6000 using KAPA HRM FAST PCR kit. The reaction mixture in a 20 μl final volume contained; 1× KAPA HRM FAST master mix, 2.5 mM MgCl_2_, 200–400 nM forward primer, 200–400 nM reverse primer, 5 ng of genomic DNA and PCR grade water. The cycling and melting conditions for EGFR exons 18 to 21 were as follow; one cycle of 95°C for 15 min; 50–70 cycles of 95°C for 10 s, 65°C for 10 s with an initial 10 cycles of touchdown (1°C/cycle), 72°C for 30 s; one cycle of 97°C for 1 min and a melt from 70°C to 95°C rising 0.2°C per second [20]. All samples were tested in duplicates.

Primer sequences that yield shorter amplicons were used in HRM assays. The sequences, retrieved from Do et al. (2008), are shown in Table 1. Primer for exon 20 was divided into two fragments (20a and 20b) to avoid the *exonic* SNP, c.2361G>A, because melting pattern arising from the SNP could not be readily extinguished from mutation by HRM.

**Table 1:** HRM primer sequences

**Exon**	**Primer name**	**Sequence**	**Amplicon size**
18	EGFR_ex18_F	5′-CATGGTGAGGGCTGAGGTGA-3′	199 bp
	EGFR_ex18_R	5′-CCAGAGG(A*)CTGTGCCAGGGAC-3′	
19	EGFR_ex19_F	5′-GTGCATCGCTGGTAACATCCA-3	250 bp
	EGFR_ex19_R	5′-AAAGGTGGGCCTGAGGTTCA-3	
20a	EGFR_ex20a_F	5′-AAGCCACACTGACGTGCCTCT-3′	121 bp
	EGFR_ex20a_R	5′-GCGTGATGAG(G*)TGCACGGT-3′	
20b	EGFR_ex20b_F	5′-CCTCCACCGTGCA(C*)CTCATC-3′	146 bp
	EGFR_ex20b_R	5′-CCCGTATCTCCCTTCCCTGA-3′	
21	EGFR_ex21_F	5′-CCTCACAGCAGGGTCTTCTCTG-3′	210 bp
	EGFR_ex21_R	5′-TGGCTGACCTAAAGCCACCTC-3′	

### HRM analysis

Data were analysed using the accompanying software (Rotor Gene). All samples were plotted according to their melting profiles. Under the difference graph, melting profiles of the samples were compared to that of the wild-type which was converted to a horizontal line. Significant deviations from the horizontal line indicate the presence of mutation and were recorded as HRM positive. HRM results were compared with results from Scorpion ARMS method to evaluate the sensitivity of the methods in EGFR mutation detection.

### Statistical analysis

Chi-square test or Fisher exact tests (SPSS version 16.0; SPSS Inc., Chicago, Ill) were used to compare *EGFR* mutation status with clinicopathologic characteristics and patient’s demographic including gender, smoking status, and ethnicity. An interrater reliability analysis using the Kappa statistic was performed to determine consistency between the two methods, Scorpion ARMS and HRM.

## Results

### Patient characteristics

From January 2011 through April 2012, a total of 484 patients with non-small cell lung cancer were tested for *EGFR* mutations. Patient’s clinical characteristics and their association with *EGFR* mutation are shown in Table 2.

**Table 2:** **Association of each variable with *EGFR *mutation**

**Characteristics**	**No.**	***EGFR *mutation No. (%)**	***P***
Patients	484	221 (45.7)	
Gender			
Male	253	81 (32.0)	<0.001
Female	231	140 (60.6)	
Missing data	0		
Age, yr			
≤ 60	244	119 (48.8)	0.163
> 60	238	101 (42.4)	
Median	60		
Range	21–92		
Missing data	2		
Ethnicity			
Chinese	293	136 (46.4)	0.747
Malay	143	62 (43.4)	
Indian	26	15 (57.7)	
Others	7	2 (28.6)	
Missing data	15	6 (40.0)	
Stage			
I/ II	5	1 (20.0)	0.385
III/ IV	72	33 (45.8)	
Not specified	348	160 (46.0)	
Missing data	59		
Histological type			
Adenocarcinoma	467	220(47.1)	0.004
Squamous cell carcinoma	12	1 (8.3)	
Large cell carcinoma	3	0 (0)	
Others	2	0 (0)	
Missing data	0		
Degree of differentiation			
Well differentiated	2	2 (100)	0.074
Moderately differentiated	42	20 (47.6)	
Poorly differentiated	33	10 (30.3)	
Undifferentiated	1	0 (0)	
Not specified	41	21 (51.2)	
Missing data	365		
Smoking history			
Current	10	5 (50.0)	0.191
Former	3	1 (33.3)	
Never	25	17 (68.0)	
Missing data	446	198 (44.4)	

Among these, 467 patients had adenocarcinoma, 12 patients had squamous cell carcinoma, 3 patients had large cell carcinoma, and 2 patients had adenosquamous carcinoma. Among the patients with adenocarcinoma, 220 cases were found to harbour at least 1 mutation in the *EGFR* gene. And in the remaining 17 non-adenocarcinoma cases, only one had an EGFR mutation, and it occurred in a female patient with squamous cell carcinoma.

Mutations in the *EGFR* gene were found in 221 of 484 patients (45.7%) and were more frequent in women (60.6%, p<0.001) and in patients with adenocarcinoma histology (47.1%, p=0.004). Although not statistically significant, the frequency of *EGFR* mutations was higher in Chinese (46.4%), in patients aged less than or equal to 60 years old (48.8%), in advanced stage patients (45.8%), in moderately differentiated tumour cells (47.6%), and in never smokers (68%). Median age of patients was 60 (range, 21–92).

### EGFR mutations detection by Scorpion ARMS

**Figure 1:**
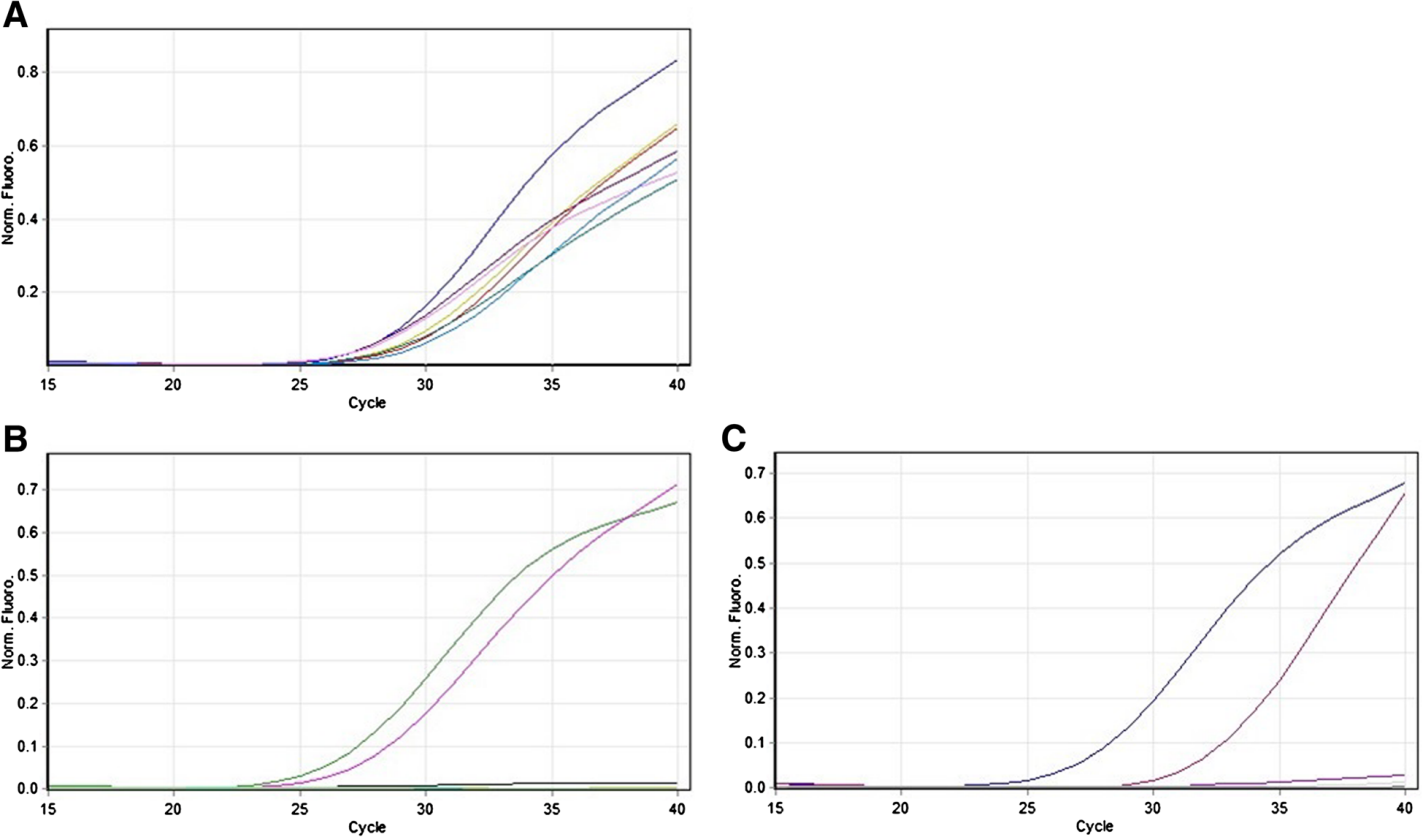
**Amplification plots from Scorpion ARMS assay. Panel A**: Amplification plots of EGFR positive controls. **Panel B**: Amplification plot showing the presence of deletions in sample 408. **Panel C**: Amplification plot showing the presence of L858R mutation in sample 429.

*EGFR* mutations were detected in 221 patients (45.7%) (Figure 1). The most common mutation was deletions in exon 19, comprising 58.9% (134/231) of all mutations found, followed by 33.8% (78/231) exon 21 mutation. Thirteen mutations in exon 20 (5.6%) and four mutations in exon 18 (1.7%) were also detected (Table 3). Interestingly, all mutations in exon 18 were found in the presence of another mutation.

**Table 3:** **Summary of *EGFR *mutations detected by Scorpion ARMS**

**Exon**	**Mutations**	**Frequency**	**Percentage (%)**
18	G719X	4	1.7
19	Deletions	134	58.0
20	S768I	13	5.6
21	L858R/ L861Q	80	34.6
	**Total**	231	100

Complex mutation patterns were also detected in the patients. Eight double mutations and one triple mutation were detected in nine patients giving rise to 231 mutations in 221 patients. Among them, five patients had the classical mutation pattern of deletions or *L858R*. Two concurrent deletions and *L858R* mutations were also observed (Table 4).

**Table 4:** **Frequency of *EGFR *mutations in different gender**

**Mutations**	**No. of samples**	**Male**	**Female**
Exon 19 deletions	128	50	78
Exon 20 insertions	5	2	3
Exon 20 S768I	2	1	1
Exon 21 L858R	74	23	51
Exon 21 L861Q	3	1	2
Exon 18 G719X + Exon 19 deletions	1	0	1
Exon 18 G719X + Exon 20 S768I	2	1	1
Exon 18 G719X + Exon 21 L861Q	1	1	0
Exon 19 deletions + Exon 20 insertions	3	1	2
Exon 19 deletions + Exon 21 L858R	1	0	1
Exon 19 deletions + Exon 20 insertions + Exon 21 L858R	1	1	0
**Total**	221	81	140

### EGFR mutation detection by HRM analysis

**Figure 2:**
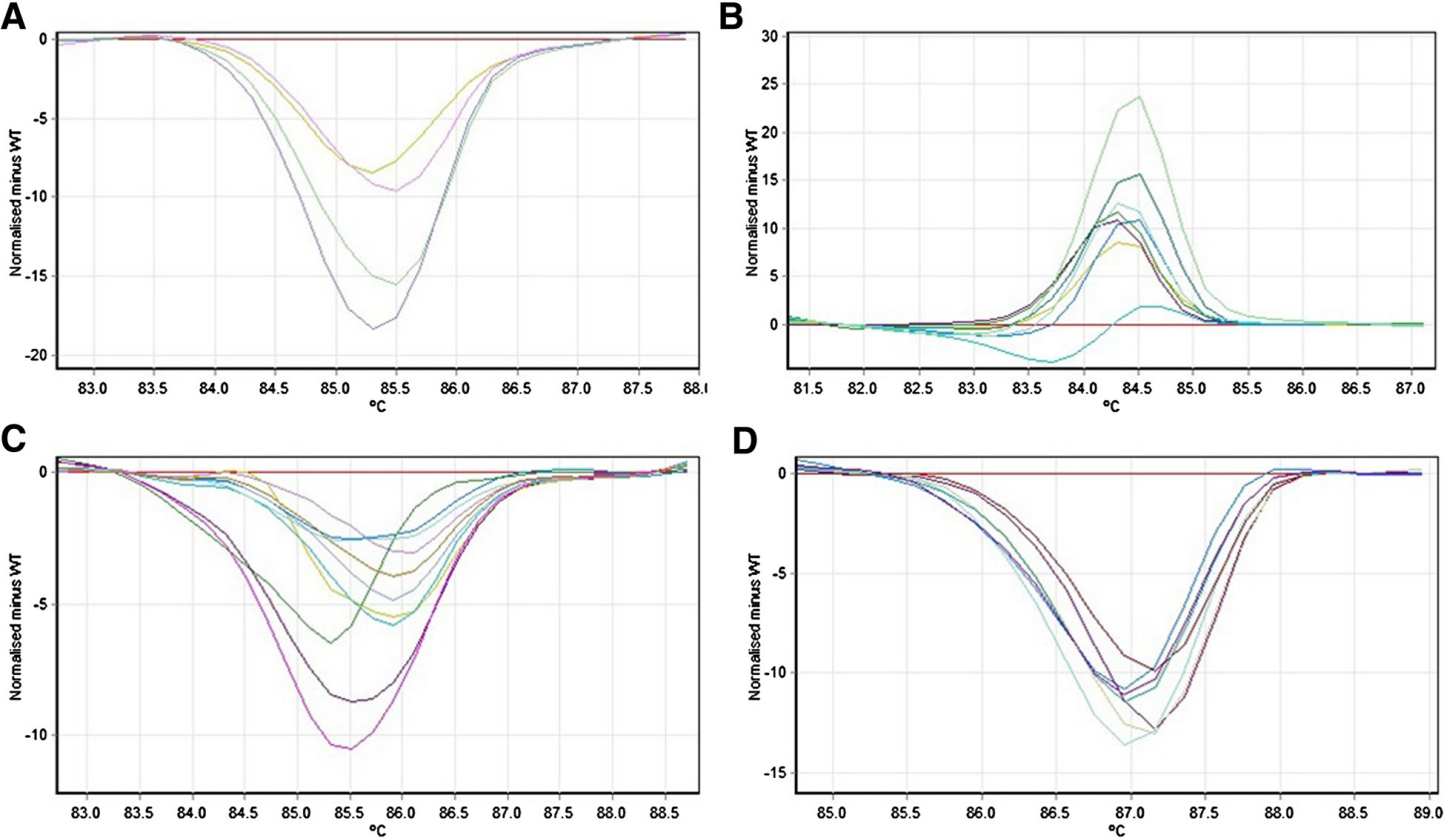
**Difference plots of EGFR exons 18–21. Panel A**: The difference plot of EGFR exon 18 shows melting profile for four positive samples (S162 in yellow, S455 in pink, S49/12 in green and S87/12 in blue). **Panel B**: The difference plot of EGFR exon 19 shows melting profiles for seven positive samples (S101 in yellow, S047 in pink, S063 in brown, S552 in green, S065 in blue, S046 in purple and S048 in orange). **Panel C**: The difference plot of EGFR exon 20 shows melting profile for four positive samples (S13 in yellow, S19 in purple, S28 in green, S68 in pink). **Panel D**: The difference plot of EGFR exon 21 shows melting profile for seven positive samples (S105/12 in purple, S115/12 in light blue, S210/12 in brown, S211/12 in green, S212/12 in dark blue, S217/12 in orange and S220/12 in pink).

In order to demonstrate the capability of HRM assay in *EGFR* mutation detection, a total of 236 NSCLC samples were tested by HRM for the detection of *EGFR* mutations in exon 18 to 21 (Figure 2). The HRM assays yielded 19, 120, 18 and 78 results that were scored as HRM positive in exon 18 to 21 respectively. All mutations identified by SARMS were correctly identified by HRM assays except for 2 samples – one each in exon 19 and 21.

However, HRM indicated more positive samples than Scorpion ARMS for all the EGFR exons. A total of 33 samples were positive only by HRM (Table 5). There were 15, 21, 8 and 4 HRM positive only results from exons 18 to 21 respectively (Table 6). Among these, 1 sample was positive in three HRM assays, 13 samples were positive in two assays, and 19 samples were positive in a single assay.

**Table 5:** Comparison of results from scorpion ARMS and HRM assays

	**Scorpion ARMS**		
**HRM**	**Positive**	**Negative**	**Total**
Positive	186	33	219
Negative	2	15	17
Total	188	48	236

**Table 6:** **Summary of *EGFR *mutation testing by Scorpion ARMS and HRM**

**Exon**	**Scorpion ARMS positive**	**HRM positive**	**HRM positive only**
18	4	19	15
19	100	120	21
20	10	18	8
21	75	78	4

## Discussion

Currently, direct sequencing is considered the “gold standard” in nucleic acids studies, but its limited sensitivity, high cost and long turnaround time limits its practicality in diagnostic setting. To produce good quality sequencing results, tumour samples in sufficient amount and in relatively good condition are required. However, these requirements are often hard to fulfil as lung tumour samples are small and contain only a small proportion of neoplastic cells, therefore resulting in a reduced sensitivity of sequencing. Besides, special instrumentation are required to perform direct sequencing, which in turn prompted the development of alternative methods that are more sensitive, faster, easier to perform, and at a reduced cost [11,20].

High resolution melting (HRM) is an emerging technique for rapid detection of DNA sequence variation that provides enormous potential to meet clinical demands [21]. HRM involves precise monitoring of the changes in fluorescence caused by the release of an intercalating DNA dye from a double stranded DNA which is denatured by increasing temperature. This technique characterizes the melting or dissociation behaviour of double-stranded PCR products based on the transition of double stranded DNA to single stranded DNA with increasing temperature. An advantage of this technique is that it does not involve any post-PCR processing as PCR amplification and melting curve analysis are performed within the same tube, thus reducing the chances of samples contamination and cross-contamination [22]. Compared to the closed tube system of HRM, normal DNA sequencing procedure involves post-PCR processing such as gel electrophoresis and gel purification before the sequencing process, thus increasing the chances of external contamination.

In this study, we compared two methods in the detection of *EGFR* mutations in NSCLC patients. Our findings showed that *EGFR* mutations were detectable in genomic samples extracted from FFPE tissue obtained from patients with NSCLC and that both Scorpion ARMS and HRM are useful methods for detection of *EGFR* mutations. The frequency of *EGFR* mutation status detected by Scorpion ARMS was statistically significantly more frequent in women (140/233 or 63.3%) than in men (81/253 or 36.7%), and more frequent in adenocarcinomas (220/467 or 47.1%) than in other histology (1/17 or 5.9%). Previous studies revealed that *EGFR* mutations were uncommon in non-adenocarcinomas [14]. In this study, we detected an exon 19 deletion in one female patient with squamous cell carcinoma. The mutation rate (45.7%) observed in our study is in concordance with previous findings [6,23-25]. The high overall mutation rate further verifies that the *EGFR* mutations were more common in Asian population. Evidently, deletions in exon 19 and point mutations in exon 21 are the two most common drug-sensitive *EGFR* mutations seen in NSCLC. These two mutations made up of 92.7% of total *EGFR* mutations detected in this study which is in agreement with previous studies [18,19,26].

Also, we identified 9 patients (4.1%) with complex mutation patterns. Among the patients with these mutations, 4 cases were found in Chinese patients, 3 in Malay patients, 1 in Indian and 1 other ethnicity. Complex *EGFR* mutations were reported to be more common in Asian lung cancer patients. However, information about the effects of complex *EGFR* mutations on patients’ response to *EGFR* TKI was very limited. Furthermore, findings from previous studies varied between each other. Tam et al. (2009) reported that *EGFR* double mutants showed attenuated responses to gefitinib compared to single classical mutations [27]. On the contrary, a study by Wu et al. (2008) showed that patients with complex *EGFR* mutations with the classical mutation pattern response better to gefitinib than those without the classical mutation pattern. Also, these patients showed longer progression free survival and overall survival times after receiving the therapy [13].

Based on the results from Scorpion ARMS, we selected a total of 236 samples, consisting both *EGFR* positive and negative mutation status, and performed HRM assays on these samples to assess its capability in detecting *EGFR* mutation as precision in identifying mutations is the fundamental in all mutation scanning methods. This study shows the reliability of HRM analysis in *EGFR* mutation detection in a panel of selected samples. All mutations identified by Scorpion ARMS were correctly identified in HRM analysis except for 2 samples – one from exon 19 assay and another from exon 21 assay. One possible explanation for this is the low level of mutation in the samples which were beyond the limit of HRM detection. The Scorpion ARMS and results from HRM assays agreed that 186 of 236 were positive and 15 were negative. The interrater reliability for the methods was found to be statistically significant with a value of Kappa = 0.40 (p <.0.001) which indicates fair agreement between the two methods [28].

One of the drawbacks of extracting DNA from FFPE material is the low yield. Thus, additional PCR cycles were needed to achieve sufficient amplification. In a study by Do et al. (2008), it was found that insufficient amplification causes a right shift of the melting curves relative to the wild-type curves in normalized plots. And by increasing the amplification cycle number to 60, the melting curves were corrected and can be reliably compared to the wild-type during analysis [20]. Depending on the degree of DNA degradation, the amount of amplifiable templates varies in each sample although all were adjusted to the same concentration (5 ng/μl).

## Conclusions

In conclusion, both Scorpion ARMS and HRM were successfully performed on genomic samples extracted from FFPE tumours. Overall, HRM compares well with the Scorpion ARMS kit. HRM indicated more positive samples than Scorpion ARMS for all the *EGFR* exons. Nevertheless, it remains to be determined whether these results were true mutation or merely false positive.

## Competing interests

The authors declare that they have no competing interests.

## Authors’ contributions

CYK designed the project, and gave critical evaluation of the manuscript; PR validated all pathological slides; TNSY performed research, analyzed the data, and draft the paper. All authors read and approved the final manuscript.
